# A Major Hindrance in Antibody Affinity Maturation Investigation: We Never Succeeded in Falsifying the Hypothesis of Single-Step Selection

**DOI:** 10.3389/fimmu.2014.00237

**Published:** 2014-05-26

**Authors:** Michal Or-Guil, Jose Faro

**Affiliations:** ^1^Systems Immunology Laboratory, Department of Biology, Humboldt-Universität zu Berlin, Berlin, Germany; ^2^Research Center ImmunoSciences, Charité-Universitätsmedizin Berlin, Berlin, Germany; ^3^Area of Immunology, Faculty of Biology, Biomedical Research Center (CINBIO), Universidade de Vigo, Vigo, Spain; ^4^Instituto Biomédico de Vigo, Vigo, Spain; ^5^Instituto Gulbenkian de Ciência, Oeiras, Portugal

**Keywords:** antibody affinity maturation, somatic hypermutation, *V* gene sequences, phylogenetic trees, selection mechanism

## Introduction

Antibody (Ab) affinity maturation (AAM) referred originally to the observed increase in average Ab affinity against a hapten (1). Later, it was found that AAM is associated with the formation of transient lymphoid structures in the B cell zones of lymphoid tissues, called germinal centers (GC), during T-cell dependent immune responses in higher vertebrates (2).

In another line of research, AAM was related to the occurrence of mutations in the variable (*V*) domain of Ab heavy (*H*) and light (*L*) chains, respectively, *V_H_* and *V_L_*. In those works, a mutational analysis of Ab *V* genes was performed, initially on bulk splenic plasma B cells and later on GC B cells vs. extrafollicular B cells, after successive immunizations. The results showed typically an increased number of mutated GC B cells (3–6), and an accumulation of mutations per Ab chain during the ongoing immune response, with many mutated B cells displaying higher affinity for the hapten used for immunization. This provided strong support to a previously suggested concept (7), according to which AAM is a B-cell receptor (BCR)-based Darwinian evolutionary process.

A few years later, two complementary hypotheses were proposed. The first one, based on a mathematical model, suggested that, for the fastest production of high affinity Abs, the mutation rate in GC B cells should be minimal before GCs reach a threshold size, and then switch abruptly to the maximal possible rate (8). The second hypothesis proposed, for the assumed Darwinian process, alternating cycles of B cell proliferation plus mutation plus selection (9). These ideas were soon extended in another modeling work, showing that Ab affinity can be maximized when the mutational mechanism switches on and off regularly (10). These results contributed considerably to strengthen the general belief in the recycling or multiple-step selection hypothesis. On the other hand, more recently, alternative B cell selection mechanisms were proposed that do not require multiple-step selection in order to be compatible with observed levels of Ab affinity increase during a primary immune response (11, 12).

There is still much to learn about AAM mechanisms (13–17), and there is a need to clarify some aspects of the GC physiology where overinterpretation and preconceptions prevail (18, 19). The multiple-step selection hypothesis is a prominent example of a concept that, having important basic and practical implications, has never been confirmed. Clearly, a direct way to establish it would be to observe multiple BCR-mediated selection events by tracking individual B cells via imaging of lymphatic tissue, observing SHM taking place between selection rounds. However, direct observation of even one selection event is not yet possible. At the same time, attempts to interpret indirect data must be faulty due to the need to use unverified assumptions on AAM mechanisms.

**Figure 1:**
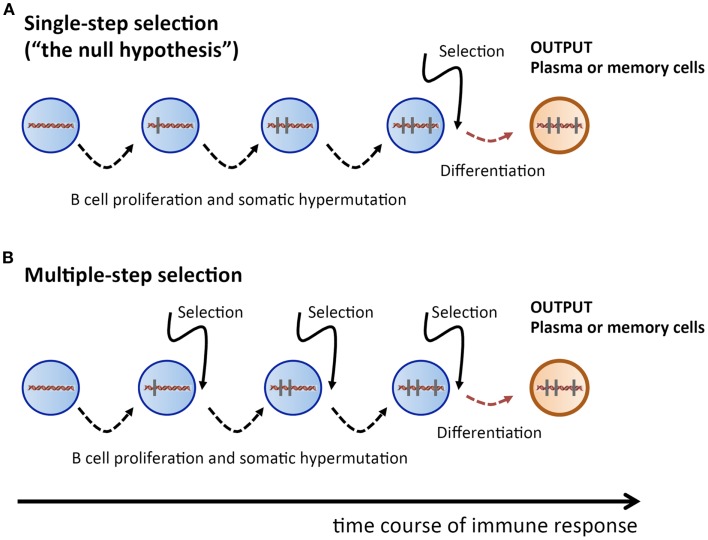
**Sketch of proliferation plus SHM and selection history of a plasma or memory cell**. **(A)** Single-step selection. Several cell division plus mutation cycles and a single final selection step before terminal differentiation into a plasma or memory cell. **(B)** Multiple-step selection. Several alternating rounds of cell division plus mutation and selection [corresponding to several rounds of **(A)**], followed by terminal differentiation into a plasma or memory cell after the last selection step. Vertical bars indicate mutations.

Therefore, we take here a radically different approach: we propose to consider the single-step selection concept to be a null-hypothesis which should be attempted to be falsified (Figure 1). Because this ansatz puts the focus on a process of random non-directed acquisition of mutations, it minimizes the need for unverified assumptions. And because mutations carry the signature of the selection process, the data to be used should consist of Ab *V* gene sequences. In the following, we examine two possible falsifying strategies.

## Falsifying the Null-Hypothesis with Phylogenetic Trees

Let us consider all mutated *V_H_* or *V_L_* sequences belonging to a given B cell lineage. The corresponding phylogenetic tree is a result of the evolutionary process undergone by the initial sequence, and as such, is shaped by the various factors pertaining to the affinity maturation process.

Extensive work was performed on developing methods to build phylogenetic trees from *V* genes of a common lineage (20) and to analyze how shape measures depend on AAM mechanisms (21, 22). These simulations show that the tree shapes vary most on the initial clone affinity and the selection threshold, and much less in dependence on the rates of GC B cell recycling (22), not allowing for a unique mapping from tree shapes to selection mechanisms – likely because the investigated trees were small. In addition to global measures not always being helpful in pointing to mechanisms at the micro-evolutionary scale (15, 23, 24), simulation of global measures like peak total GC B cell numbers did not lead to results that contradict the single-step hypothesis (22).

Summing up, the null-hypothesis has never been falsified by examining the shapes of phylogenetic trees.

## Falsifying the Null-Hypothesis by Counting Recurrent Mutations

Let us consider a thought experiment, in which two syngeneic mice with a single, non-mutated B cell clone expressing the same *V* genes, are immunized with the same antigen, and that both mice initiate a process of AAM in which the selection forces acting on the diversified *V* sequences are identical. Let us further assume that the baseline mutability during SHM is uniformly distributed along rearranged *V* genes and independent on the time elapsed after immunization. After a number of days, a sample of Ab *V* genes is sequenced and an *independent set of V sequences* is obtained for each mouse. As a result of the stochastic nature of SHM, the mutation distribution in both sets may be quite different. If nevertheless an identical set of mutations appears in both independent data sets, we call it a *recurrent mutation pattern*.

How likely is it to find a recurrent mutation pattern? Assuming that the AAM process in our mice followed a single-step selection scheme (Figure 1A), we can make a first rough estimation. Consider the probability $p_{k}^{L}$ to obtain a particular pattern of *k* mutations out of all possible patterns of *k* mutations, which is $p_{k}^{L}=\frac{1}{M_{k}^{L}}$, with $M_{k}^{L}=3^{k}\times {(}_{K}^{L})$ being the number of possible mutation patterns of size *k*, and *L* being the *V* sequence length. When an Ab *V* gene of length *L* = 300 and *k* mutations is produced by the SHM process, the probability that the outcome is a particular mutation pattern is $p_{1}^{300}\approx 10^{-3}$ for *k* = 1, and $p_{2}^{300}\approx 10^{-6}$ for *k* = 2. During an AAM process, thousands of mutated B cells are generated in a mouse; hence, the probability of finding a given mutation pattern of size *k* among all mutated B cells is $1-{\left(1-p_{k}^{300}\right)}^{N}$, where *N* is the number of B cells with *k* mutations. Let us assume that *N* = 10^5^ B cells got *k* = 2 mutations. Then, as a crude estimation, the probability of a given mutation pattern of size *k* = 1 and *k* = 2 among all those B cells is, respectively, $1-{\left(1-p_{1}^{300}\right)}^{10^{5}}\approx 1$ and $1-{\left(1-p_{2}^{300}\right)}^{10^{5}}\approx 0.1$. This means that recurrent mutation patterns of such a small size are rather likely to appear in a single-step setting, and are therefore not suitable to contradict the null-hypothesis.

However, for *k* = 5, the probability of obtaining a particular mutation pattern by chance among 10^5^ B cells is only $1-{\left(1-p_{5}^{300}\right)}^{10^{5}}\approx 2\times 10^{-8}$. Hence it is highly improbable that both mice in our thought experiment could produce, by a single-step process, the same recurrent mutation pattern. On the other hand, in a multiple-step selection process, single mutations can be selected one by one (Figure 1B). Therefore, finding recurrent mutation patterns of that size or larger would be consistent with a multiple-step scheme while deeming the single-step null-hypothesis highly improbable. Admittedly, the above is a simplified probability calculation. A more realistic estimation, based on calculations that include reversions and different baseline mutabilities, does not change the above conclusions (see Supplementary Material).

Data from experiments along the main idea of our thought experiment do exist. For instance, Rag1^−/−^ double transgenic mice for Ab *H* and *L* chains are available (25). Also, hapten-conjugated proteins can yield a large percentage of canonical *V* gene sequences (3).

In a survey, we found a number of publications that present Ab *V* sequences obtained from syngeneic mice under the same immunization protocol (3, 4, 26–31). In all the data analyzed so far we could not find a single instance of mutated *V* sequences from GC B cells sharing three or more mutations. Also, a substantial set of independent murine *V_H_* genes with a common *V_H_* germline sequence was recently collected from literature and examined for recurrent patterns (32). The search yielded not a single case of shared triplets.

In summary, to our knowledge, there is no published independent sequence data that contradicts the single-step hypothesis.

## Challenge for Future Research: Trying to Falsify the Single-Step Hypothesis with High-Throughput Sequence Data

A clear understanding of AAM requires answering the question whether the single-step or the multiple-step selection hypothesis hold. A straightforward approach would be direct observation of SHM and Ab-mediated selection events via *in vivo* imaging, but this is technically not yet possible. Similarly daunting is to try to infer the frequency of selection steps from indirect observations while making use of non-validated assumptions.

Our proposal of falsifying the single-step null-hypothesis provides a way out. This hypothesis does not preclude the knowledge of any mechanisms besides the stochastic process of SHM. Moreover, this knowledge does not need to be highly precise because an *upper* estimation of probabilities under the null-hypothesis can suffice.

Therefore, examining Ab *V* gene sequence data with the aim of falsifying the single-step hypothesis is a powerful technique.

Next generation sequencing currently allows to obtain suitable Ab *V* gene sequence data (33, 34). One strategy for the search of contradictions is to calculate, under the null-hypothesis, the probability distribution of recurrent mutation patterns acquired independently.

A detailed calculation of probability distributions is shown in the Supplementary Material. It allows to estimate that, for given realistic parameters (see Table therein), the probabilities of observing recurrent patterns are much lower than 0.05. In case of a very strict multiple-step selection, the null-hypothesis can potentially be contradicted with very few sequences.

This strategy can be pursued both for independent and same-lineage *V* gene sequence sets. In the latter case, the probability calculation must be performed exclusively for recurrent patterns that cannot possibly stem from common ancestors.

Another strategy comprises trying to contradict the null-hypothesis by examining the structure of a same-lineage *V* sequence population for signs of a directed multi-step process as in contrast to an undirected, random process. Such signs can be, for instance, the emergence of independent quasi-species (35), or of coalescence times typical to multi-step processes (36).

These methods require however: (i) that the AAM process has been ongoing long enough for population structures to have emerged, and (ii) that enough sequences can be retrieved to make these structures visible. It is well possible that times are too short and clonal sizes too small to provide this sort of data.

No matter which strategy turns out to be the best, important challenges are still open. For instance, present methods of calculating the pairwise probability that sequences pertain to a common or to a different B cell lineage (32) need to be improved, especially where short junctional regions make identification of lineage difficult. With such an analysis working, different independent Ab *V* sequence sets can also be retrieved from the same individual. A further challenge consists of devising estimators of the recurrent mutation pattern probability distributions adequate to the respective experimental setup. Good estimations of baseline mutability would be helpful; however, using upper estimations of probability might be sufficient.

For pinning down the actual AAM process, it is not advisable to examine data sets that include sequences of memory cells, to avoid the risk of analyzing repeated rounds of immunizations against the same or different antigens. Thus, the design of experiments that consider both the anatomical compartment from which B cells are taken and strategies that maximize the size of data sets, poses a challenge as well.

While multiple-step selection points to AAM as an accelerated molecular evolution process maximizing Ab affinity increase, single-step selection points at an optimization process of Ab repertoires in which both Ab affinity enhancement and diversification can be equally relevant (14, 17, 37). Striving to discover which is right must be a priority to those interested in unveiling AAM mechanisms. Trying to falsify the single-step hypothesis is not easy and might be even impossible – for instance, if the underlying process is indeed a single-step one. But it is, in our opinion, the only viable way.

## Conflict of Interest Statement

The authors declare that the research was conducted in the absence of any commercial or financial relationships that could be construed as a potential conflict of interest.

## Supplementary Material

The Supplementary Material for this article can be found online at http://www.frontiersin.org/Journal/10.3389/fimmu.2014.00237/full

## References

[1] Eisen HN Siskind GW Variations in affinities of antibodies during the immune response. Biochemistry (1964) 3:996–1008 10.1021/bi00895a027 14214095

[2] Flajnik MF Comparative analyses of immunoglobulin genes: surprises and portents. Nat Rev Immunol (2002) 2(9):688–98 10.1038/nri889 12209137

[3] Berek C Berger A Apel M Maturation of the immune response in germinal centers. Cell (1991) 67(6):1121–9 10.1016/0092-8674(91)90289-B 1760840

[4] Jacob J Kelsoe G Rajewsky K Weiss U Intraclonal generation of antibody mutants in germinal centres. Nature (1991) 354(6352):389–92 10.1038/354389a0 1956400

[5] Griffiths GM Berek C Kaartinen M Milstein C Somatic mutation and the maturation of immune response to 2-phenyl oxazolone. Nature (1984) 312(5991):271–5 10.1038/312271a0 6504141

[6] Berek C Milstein C Mutation drift and repertoire shift in the maturation of the immune-response. Immunol Rev (1987) 96:23–41 10.1111/j.1600-065X.1987.tb00507.x 3298007

[7] MacLennan IC Gray D Antigen-driven selection of virgin and memory B cells. Immunol Rev (1986) 91:61–85 10.1111/j.1600-065X.1986.tb01484.x 3089914

[8] Agur Z Mazor G Meilijson I Maturation of the humoral immune response as an optimization problem. Proc Biol Sci (1991) 245(1313):147–50 10.1098/rspb.1991.0101 1682938

[9] MacLennan IC Johnson GD Liu YJ Gordon J The heterogeneity of follicular reactions. Res Immunol (1991) 142(3):253–7 10.1016/0923-2494(91)90070-Y 1896616

[10] Kepler TB Perelson AS Somatic hypermutation in B cells: an optimal control treatment. J Theor Biol (1993) 164(1):37–64 10.1006/jtbi.1993.1139 8264243

[11] Moreira JS Faro J Re-evaluating the recycling hypothesis in the germinal centre. Immunol Cell Biol (2006) 84(4):404–10 10.1111/j.1440-1711.2006.01443.x 16834575

[12] Raoof S Heo M Shakhnovich EI A one-shot germinal center model under protein structural stability constraints. Phys Biol (2013) 10(2):025001 10.1088/1478-3975/10/2/025001 23492682 PMC4777297

[13] Manser T Textbook germinal centers? J Immunol (2004) 172(6):3369–75 10.4049/jimmunol.172.6.3369 15004133

[14] Longo NS Lipsky PE Why do B cells mutate their immunoglobulin receptors? Trends Immunol (2006) 27(8):374–80 10.1016/j.it.2006.06.007 16809065

[15] Or-Guil M Wittenbrink N Weiser AA Schuchhardt J Recirculation of germinal center B cells: a multilevel selection strategy for antibody maturation. Immunol Rev (2007) 216:130–41 10.1111/j.1600-065X.2007.00507.x 17367339

[16] Allen CD Okada T Cyster JG Germinal-center organization and cellular dynamics. Immunity (2007) 27(2):190–202 10.1016/j.immuni.2007.07.009 17723214 PMC2242846

[17] Faro J Combadao J Gordo I Did germinal centers evolve under differential effects of diversity vs affinity? In: Bersini H Carneiro J, editors. Artificial Immune Systems: 5th International Conference, ICARIS 2006, Oeiras, Portugal, September 4-6, 2006: Proceedings. Lecture Notes in Computer Science New York, NY: Springer (2006). p. 1–8

[18] Faro J Or-Guil M Reassessing germinal centre reaction concepts. In: Molina-París C Lythe G, editors. Mathematical Models and Immune Cell Biology. New York, NY: Springer (2011). p. 241–58

[19] Faro J Or-Guil M How oligoclonal are germinal centers? A new method for estimating clonal diversity from immunohistological sections. BMC Bioinformatics (2013) 14(Suppl 6):S8 10.1186/1471-2105-14-S6-S8 23734629 PMC3633029

[20] Barak M Zuckerman NS Edelman H Unger R Mehr R IgTree: creating immunoglobulin variable region gene lineage trees. J Immunol Methods (2008) 338(1–2):67–74 10.1016/j.jim.2008.06.006 18706908

[21] Dunn-Walters DK Belelovsky A Edelman H Banerjee M Mehr R The dynamics of germinal centre selection as measured by graph-theoretical analysis of mutational lineage trees. Dev Immunol (2002) 9(4):233–43 10.1080/10446670310001593541 15144020 PMC2276115

[22] Shahaf G Barak M Zuckerman NS Swerdlin N Gorfine M Mehr R Antigen-driven selection in germinal centers as reflected by the shape characteristics of immunoglobulin gene lineage trees: a large-scale simulation study. J Theor Biol (2008) 255(2):210–22 10.1016/j.jtbi.2008.08.005 18786548

[23] Wittenbrink N Weber TS Klein A Weiser AA Zuschratter W Sibila M Broad volume distributions indicate nonsynchronized growth and suggest sudden collapses of germinal center B cell populations. J Immunol (2010) 184(3):1339–47 10.4049/jimmunol.0901040 20053939

[24] Wittenbrink N Klein A Weiser AA Schuchhardt J Or-Guil M Is there a typical germinal center? A large-scale immunohistological study on the cellular composition of germinal centers during the hapten-carrier-driven primary immune response in mice. J Immunol (2011) 187(12):6185–96 10.4049/jimmunol.1101440 22102720

[25] Paus D Phan TG Chan TD Gardam S Basten A Brink R Antigen recognition strength regulates the choice between extrafollicular plasma cell and germinal center B cell differentiation. J Exp Med (2006) 203(4):1081–91 10.1084/jem.20060087 16606676 PMC2118299

[26] Kallberg E Gray D Leanderson T Kinetics of somatic mutation in lymph node germinal centres. Scand J Immunol (1994) 40(5):469–80 10.1111/j.1365-3083.1994.tb03492.x 7973453

[27] Ziegner M Steinhauser G Berek C Development of antibody diversity in single germinal centers: selective expansion of high-affinity variants. Eur J Immunol (1994) 24(10):2393–400 10.1002/eji.1830241020 7925566

[28] Kimoto H Nagaoka H Adachi Y Mizuochi T Azuma T Yagi T Accumulation of somatic hypermutation and antigen-driven selection in rapidly cycling surface Ig+ germinal center (GC) B cells which occupy GC at a high frequency during the primary anti-hapten response in mice. Eur J Immunol (1997) 27(1):268–79 10.1002/eji.1830270140 9022029

[29] Jacob J Przylepa J Miller C Kelsoe G In situ studies of the primary immune response to (4-hydroxy-3-nitrophenyl)acetyl. III. The kinetics of V region mutation and selection in germinal center B cells. J Exp Med (1993) 178(4):1293–307 10.1084/jem.178.4.1293 8376935 PMC2191212

[30] Han S Zheng B Dal Porto J Kelsoe G In situ studies of the primary immune response to (4-hydroxy-3-nitrophenyl)acetyl. IV. Affinity-dependent, antigen-driven B cell apoptosis in germinal centers as a mechanism for maintaining self-tolerance. J Exp Med (1995) 182(6):1635–44 10.1084/jem.182.6.1635 7500008 PMC2192250

[31] Vora KA Tumas-Brundage K Manser T Contrasting the in situ behavior of a memory B cell clone during primary and secondary immune responses. J Immunol (1999) 163(8):4315–27 10510371

[32] Weiser AA Wittenbrink N Zhang L Schmelzer AI Valai A Or-Guil M Affinity maturation of B cells involves not only a few but a whole spectrum of relevant mutations. Int Immunol (2011) 23(5):345–56 10.1093/intimm/dxr018 21521882

[33] Benichou J Ben-Hamo R Louzoun Y Efroni S Rep-Seq: uncovering the immunological repertoire through next-generation sequencing. Immunology (2012) 135(3):183–91 10.1111/j.1365-2567.2011.03527.x 22043864 PMC3311040

[34] Liberman G Benichou J Tsaban L Glanville J Louzoun Y Multi step selection in Ig H chains is initially focused on CDR3 and then on other CDR regions. Front Immunol (2013) 4:274 10.3389/fimmu.2013.00274 24062742 PMC3775539

[35] Eigen M Selforganization of matter and the evolution of biological macromolecules. Naturwissenschaften (1971) 58(10):465–523 10.1007/BF00623322 4942363

[36] Brunet E Derrida B Genealogies in simple models of evolution. J Stat Mech (2013) 2013:P01006 10.1088/1742-5468/2013/01/P01006

[37] Baumgarth N How specific is too specific? B-cell responses to viral infections reveal the importance of breadth over depth. Immunol Rev (2013) 255(1):82–94 10.1111/imr.12094 23947349 PMC3748619

