# Supplementary material for: A Major Hindrance in Antibody Affinity Maturation Investigation: We Never Succeeded in Falsifying the Hypothesis of Single-Step Selection
Source: Front Immunol. 2014 May 26;5:237. doi: 10.3389/fimmu.2014.00237 (PMC4033600; doi:10.3389/fimmu.2014.00237)
Supplement: Supplementary file 1 [file Data_Sheet1.PDF]

Supplementary Information

The null hypothesis states that several cell division cycles and SHM take place before a single selection round generates the output B cells (memory B cells and plasma cells). This means that all mutational patterns in the output mutant B cells must have been previously generated either randomly or with the only biases intrinsic to the SHM machinery. The aim in this appendix is to determine the probability P_{share} that in two independent V sequence samples, A and B, with sizes n_A and n_B, respectively, at least one recurrent (common) mutation pattern of size k is present. The assumptions we make here with respect to the dynamics of dividing B cells undergoing affinity maturation are: 1) there is a single selection step (null hypothesis), 2) sampled B cells have undergone g division cycles since onset of SHM. With respect to the mutation probability, we assume it is uniform, constant in time, and equal and independent in both daughter cells, so that any nucleotide can mutate to any of the other three different nucleotides with probability p. Thus, $3p$ is the probability of mutating at a given base pair per cell cycle (for simplicity, we consider the probabilities of transversions and transitions to be equal), and $q = 1 - 3p$ the probability of not mutating at a given base pair per cell cycle.

Probability of finding multiple mutations after g division cycles

Let consider V gene sequences have L base pairs. In the first cell division the probability of a cell to get m mutations in a V gene is:

$$\binom{L}{m}(1-q)^m\, q^{L-m}. \tag{1}$$

And the probability that a cell acquires m mutations in a V gene after g division cycles, is given by the following expression:

$$\begin{aligned} P_g(L,\, m) &= \sum_{s=0}^{L} P_{g-1}(L,\, s) \sum_{i=0}^{s} \binom{L-s}{m-i}(1-q)^{m-i}\, q^{L-s-(m-i)} \binom{s}{i}(1-p)^i p^{s-i} \\ &= (1-q)^m\, q^{L-m} \sum_{i=0}^{m} \sum_{s=i}^{L-m+i} P_{g-1}(L,\, s)\binom{L-s}{m-i}\binom{s}{i}\left(\frac{1-p}{1-q}\right)^i \left(\frac{p}{q}\right)^{s-i}. \end{aligned} \tag{2}$$

This recursive formula takes into account the possibility of mutation of a mutated nucleotide to the original nucleotide (reversion) or to a different one and corresponds to the case investigated by Jukes and Cantor [1]. We can use induction to obtain an explicit expression. Let us assume that,

$$P_g(L, m) = \binom{L}{m}\Big((1-q)\,A_g\Big)^m \Big(1-(1-q)\,A_g\Big)^{L-m}, \tag{3}$$

where $A_g = \sum_{i=0}^{g-1}(q-p)^i$, and consider the equality:

$$\binom{L}{s}\binom{s}{i}\binom{L-s}{m-i} = \binom{L}{m}\binom{m}{i}\binom{L-m}{s-i}. \tag{4}$$

Then,

$$\begin{aligned}
P_{g+1}(L, m) &= (1-q)^m\, q^{L-m} \sum_{i=0}^{m}\sum_{s=i}^{L-m+i} P_g(L, s)\binom{L-s}{m-i}\binom{s}{i}\left(\frac{1-p}{1-q}\right)^i\left(\frac{p}{q}\right)^{s-i} \\
&= (1-q)^m\, q^{L-m} \sum_{i=0}^{m}\sum_{s=i}^{L-m+i} \binom{L}{s}\Big((1-q)\,A_g\Big)^s\Big(1-(1-q)\,A_g\Big)^{L-s}\binom{L-s}{m-i}\binom{s}{i}\left(\frac{1-p}{1-q}\right)^i\left(\frac{p}{q}\right)^{s-i} \\
&= (1-q)^m\, q^{L-m}\binom{L}{m}\sum_{i=0}^{m}\binom{m}{i}\left(\frac{1-p}{1-q}\right)^i\left(\frac{(1-q)\,A_g}{1-(1-q)\,A_g}\right)^i\sum_{s=i}^{L-m+i}\left(\frac{(1-q)\,A_g}{1-(1-q)\,A_g}\right)^{s-i}\binom{L-m}{s-i}\left(\frac{p}{q}\right)^{s-i} \\
&= \binom{L}{m}(1-q)^m\, q^{L-m}\Big(1-(1-q)\,A_g\Big)^L\left(1+\frac{(1-p)\,A_g^i}{1-(1-q)\,A_g}\right)^m\left(1+\frac{p(1-q)\,A_g}{q\Big(1-(1-q)\,A_g\Big)}\right)^{L-m} \\
&= \binom{L}{m}\Big((1-q)\,A_{g+1}\Big)^m\Big(1-(1-q)\,A_{g+1}\Big)^{L-m}.
\end{aligned} \tag{5}$$

Since $P_0(L, 0) = 1$ and $P_0(L, m) = 0$, for $m > 0$, then Eqn. 3 is correct. Moreover, given that $A_g = \sum_{i=0}^{g-1}(q-p)^i = \frac{1-(q-p)^g}{1-(q-p)}$, it follows that the probability that a random sequence has exactly m mutations is,

$$P_g(L, m) = \binom{L}{m}\left((1-q)\,\frac{\Big(1-(q-p)^g\Big)}{1-(q-p)}\right)^m\left(1-(1-q)\,\frac{\Big(1-(q-p)^g\Big)}{1-(q-p)}\right)^{L-m}, \tag{6}$$

Also, the probability that a given k-plet is present in a random sequence with exactly m ($\geq k$) mutations is given by,

$$\begin{aligned} P_{k_1}(L, m) &= P_g(L, m)\,\frac{\binom{m}{k}}{3^k\binom{L}{k}} = \left((1-q)\,\frac{\left(1-(q-p)^g\right)}{1-(q-p)}\right)^m \left(1-(1-q)\,\frac{\left(1-(q-p)^g\right)}{1-(q-p)}\right)^{L-m} \binom{L}{m}\frac{\binom{m}{k}}{3^k\binom{L}{k}} \\ &= \left((1-q)\,\frac{\left(1-(q-p)^g\right)}{1-(q-p)}\right)^m \left(1-(1-q)\,\frac{\left(1-(q-p)^g\right)}{1-(q-p)}\right)^{L-m} \frac{1}{3^k}\binom{L-k}{m-k}, \end{aligned} \tag{7}$$

where $\binom{m}{k}$ is the number of different k-plets that can be formed in a sequence with m ($\geq k$) mutations, and $N_{Lk} = \frac{\binom{m}{k}}{3^k\binom{L}{k}}$ is the probability of a given k-plet within a random sequence with m ($\geq k$) mutations.

Finally, the probability of a given k-plet in a random sequence is:

$$P_{L,k} = \sum_{m=0}^{L} P_{k_1}(L, m). \tag{8}$$

Tipically, researchers have sampled GC B-cell H or L sequences containing a given V_H or V_L gene or any V gene of a given family, independently of $D_H J_H$ or J_L sequences. Let consider now two independent sequence sets of those, A and B. The probability that a random sequence from a sample A has not any of the k-plets in n_B sequences in another sample B is:

$$(1 - P_{L,k})^{T^{diff}_{n_B}}, \tag{9}$$

where $T^{diff}_{n_B}$ is the total number of different k-plets in the n_B sequences.

Therefore, the probability that the n_A sequences from sample A has not any of the k-plets in the n_B sequences of sample B is:

$$\left((1 - P_{L,k})^{T^{diff}_{n_B}}\right)^{n_A} = (1 - P_{L,k})^{n_A \times T^{diff}_{n_B}}. \tag{10}$$

And the probability that the n_A sequences in sample A share at least one of the k-plets in the n_B sequences of sample B is:

$$P_{share} = 1 - (1 - P_{L,k})^{n_A \times T^{diff}_{n_B}}. \tag{11}$$

In order to estimate $T^{diff}_{n_B}$, consider two sequences, one with m_1 mutations (and hence, $r_1 = \binom{m_1}{k}$ k-plets) and the other with m_2 mutations (and hence, $r_2 = \binom{m_2}{k}$ k-plets). Then the probability that the two sequences share i k-plets is: $p_i(r_1, r_2) = \binom{r_1}{i}\binom{N_{Lk}-r_1}{r_2-i}\Big/\binom{N_{Lk}}{r_2}$; it is not difficult to show that $p_i(r_1, r_2) = p_i(r_2, r_1)$.

Notice also that $\sum_{i=0}^{r_1}\binom{r_1}{i}\binom{N_{Lk}-r_1}{r_2-i} = \binom{N_{Lk}}{r_2}$ (which is known as the Chu-Vandermonde identity). Hence: $\sum_{i=0}^{r_1} p_i(r_1, r_2) = \sum_{i=0}^{r_2} p_i(r_2, r_1) = 1$. Therefore, the number of k-plets expected to be shared by two random sequences, one with m_1 mutations and the other with m_2 mutations is:

$$n_k(r_1, r_2) = \sum_{i=0}^{r_1} i \times p_i(r_1, r_2), \tag{12}$$

and the total number of different k-plets among the two sequences is:

$$r_1 + r_2 - n_k(r_1, r_2). \tag{13}$$

Denote $s_1 = r_1$, and $s_2 = r_1 + r_2 - n_k(r_1, r_2) = r_1 + r_2 - n_k(s_1, r_2)$ and let consider a third sequence with r_3 k-plets. Then the number of different k-plets among those of sequences 1 and 2 and those of sequence 3 is:

$$s_3 = s_2 + r_3 - n_k(s_2, r_3) = r_1 + r_2 + r_3 - n_k(s_1, r_2) - n_k(s_2, r_3). \tag{14}$$

In general, considering n_B sequences in a sample, the number of different k-plets among all those n_B sequences is:

$$T^{diff}_{n_B} = s_{n_B} = \sum_{i=1}^{n_B} r_i - \sum_{i=1}^{n_B-1} n_k(s_i, r_{i+1}) = r_{n_B} + \sum_{i=1}^{n_B-1} (r_i - n_k(s_i, r_{i+1})). \tag{15}$$

For a more realistic scenario, where baseline mutability is not the same for all microsequences and all positions along a V gene sequence, this has to be taken into account accordingly. We have further developed the above model to include two subsets of nucleotides, L_1 and L_2 (with

$L = L_1 + L_2$), within a V sequence, with different mutation probabilities, p_1 and p_2, respectively. In this case, the probability that after g division cycles a sequence has m mutations is given by:

$$P_g(L_1, L_2, m) = \sum_{m_1=0}^{m} P_g(L_1, m_1) \times P_g(L_2, m_2). \tag{16}$$

where, $P_g(L_i, m_i)$ is the probability defined in Eqn. 6, for a V sequence of length L_i and baseline mutability p_i, with $i = 1, 2$.

Although the formulas are more involved, a closed form for $P_g(L_1, L_2, m)$ can still be obtained:

$$\begin{aligned} P_g(L_1, L_2, m) \;=\; & \left(\frac{1 + 3 \times (1 - 4p_1)^g}{4}\right)^{L_1} \times \left(\frac{1 + 3 \times (1 - 4p_2)^g}{4}\right)^{L_2} \times \left(\frac{3 \times \left(1 - (1 - 4p_2)^g\right)}{1 + 3 \times (1 - 4p_2)^g}\right)^m \times \\ & \binom{L_2}{m} \times {}_2F_1(a, b, c, z). \end{aligned} \tag{17}$$

where ${}_2F_1$ is the Gaussian hypergeometric function, with $a = -L_1$; $b = -m$; $c = 1 + L_2 - m$; and

$$z = \frac{\left(-1 + (1 - 4p_1)^g\right) \times \left(1 + 3 \times (1 - 4p_2)^g\right)}{\left(1 + 3 \times (1 - 4p_1)^g\right) \times \left(-1 + (1 - 4p_2)^g\right)} \tag{18}$$

As expected, for $p_1 = p_2$ or for $L_1 = 0$ (or $L_2 = 0$) the formula for this probability boils down to that of Eqn. 6. In Table 1, the results for the calculation of P_{share} for different values of k, p_1, and p_2 are given, corresponding to both the uniform and the non-uniform mutation probability cases, and assuming that sample sizes are $n_A = n_B = 20$, and that each sequence has the expected (average) number of k-plets.

Table 1: P_{share} for different parameter values, with $L = 300$ and $L_1 = 30^a$.

	k	P_{share}		
		$p_1 = 0.0003$ $p_2 = 0.0003$	$p_1 = 0.0003$ $p_2 = 0.00003$	$p_1 = 0.00003$ $p_2 = 0.00003$
$g = 15$	3	0.00071	3.3×10^{-8}	7.5×10^{-10}
	4	3.9×10^{-6}	6.3×10^{-12}	4.2×10^{-14}
	5	1.7×10^{-8}	9.5×10^{-16}	1.8×10^{-18}
$g = 21$	3	0.0052	2.4×10^{-7}	5.6×10^{-9}
	4	5.6×10^{-5}	9.1×10^{-11}	6.1×10^{-13}
	5	4.7×10^{-7}	2.7×10^{-14}	5.3×10^{-17}

[a]Usual estimates for the mutation probability, $\bar{p}$, are $10^{-4} - 10^{-3}$ per base pair and division cycle; notice that $p_1, p_2 = \frac{1}{3}\bar{p}$. Assuming GC B cells follow three division cycles per day, the used values $g = 15$ and $g = 21$ correspond, respectively, to 5 and 8 days after the onset of SHM. Since the SHM mechanism is estimated to be activated between days 5 and 7 of a primary immune response (see, for instance, references [2–6]) the above values for g correspond to days 10 and 12 (or 13 and 15) of the immune response.

References

[1] Jukes, T.H. and Cantor, C.R. (1969). Evolution of Protein Molecules. New York: Academic Press.

[2] Levy, N.S., Malipiero, U.V., Lebecque, S.G., and Gearhart, P.J. (1989). Early onset of somatic mutation in immunoglobulin VH genes during the primary immune response. J Exp Med, 169:2007-2019.

[3] Jacob, J., Przylepa, J., Miller, C., Kelsoe, G. (1993). In situ studies of the primary immune response to (4-hydroxy-3-nitrophenyl)acetyl. III. The kinetics of V region mutation and selection in germinal center B cells. J Exp Med, 178:1293-1307.

[4] McHeyzer-Williams, M.G., McLean, M.J., Lalor, P.A., Nossal, G.J. (1993). Antigen-driven B cell differentiation in vivo. J Exp Med, 178:295-307.

[5] Miller, C., Stedra, J., Kelsoe, G., and Cerny, J. (1995). Facultative role of germinal centers and T cells in the somatic diversification of IgVH genes. J Exp Med, 181:1319-1331.

[6] Tarlinton, D.M., Light, A., Nossal, G.J.V., And Smith, K.G.C. (1998). Affinity maturation of the primary response by V gene diversification. Curr Topics Microbiol Immunol, 229:71-83.
